# Polyolefin/ZnO Composites Prepared by Melt Processing

**DOI:** 10.3390/molecules24132432

**Published:** 2019-07-02

**Authors:** Alojz Anžlovar, Mateja Primožič, Iztok Švab, Maja Leitgeb, Željko Knez, Ema Žagar

**Affiliations:** 1 Department of Polymer Chemistry and Technology, National Institute of Chemistry, Hajdrihova 19, SI-1000 Ljubljana, Slovenia; 2 Faculty of Chemistry and Chemical Engineering, University of Maribor, Smetanova ulica 17, SI-2000 Maribor, Slovenia; 3 ISOKON d.o.o., Industrijska cesta 16, SI-3210 Slovenske Konjice, Slovenia

**Keywords:** high density polyethylene, polypropylene, ZnO nanoparticles, composites, antibacterial activity, UV-Vis stability, mechanical and thermal properties

## Abstract

Composites of polyolefin matrices (HDPE and PP) were prepared by melt processing using two commercially available nano ZnO powders (Zinkoxyd aktiv and Zano 20). The mechanical and thermal properties, UV-Vis stability, and antibacterial activity of composites were studied. Tensile testing revealed that both nano ZnO types have no particular effect on the mechanical properties of HDPE composites, while some positive trends are observed for the PP-based composites, but only when Zano 20 was used as a nanofiller. Minimal changes in mechanical properties of composites are supported by an almost unaffected degree of crystallinity of polymer matrix. All polyolefin/ZnO composites exposed to artificial sunlight for 8–10 weeks show more pronounced color change than pure matrices. This effect is more evident for the HDPE than for the PP based composites. Color change also depends on the ZnO concentration and type; composites with Zano 20 show more intense color changes than those prepared with Zinkoxyd aktiv. Results of the antibacterial properties study show very high activity of polyolefin/ZnO composites against *Staphylococcus aureus* regardless of the ZnO surface modification, while antibacterial activity against *Escherichia coli* shows only the composites prepared with unmodified ZnO. This phenomenon is explained by different membrane structure of gram-positive (*S. aureus*) and gram-negative (*E. coli*) bacteria.

## 1. Introduction

Polymer nanocomposites with inorganic nanoparticles are propulsive fields of research due to innovative combination of properties, arising from the application of inorganic nanofiller. The introduction of nanofillers into the polymer matrices, in most cases, generates relevant problems in terms of their dispersibility [1]. Since inorganic nanoparticles are mostly hydrophilic, they substantially differ in surface energy from the hydrophobic polymer matrices, causing phase segregation of both composite constituents. Nanoparticles are inclined towards formation of stable agglomerates, which are difficult to break up into individual particles and to disperse them uniformly in the host polymer matrix [2]. Due to a relatively small specific interface surface of agglomerates, they prevent efficient transfer of beneficial properties of the nanofiller, related to its nanoscopic dimension and to the host polymer, resulting in ‘nanofilled materials’ with properties similar to the traditional microcomposites [3]. In accordance with these considerations, it is clear that controlling the dispersion of nanoparticles throughout the polymer matrix is highly important to fully exploit a potential of polymer nanocomposites. In this respect, the development of effective mixing and dispersion procedure is crucial in nanocomposite preparation [4]. Various approaches have been proposed for the manufacturing of polymer nanocomposites with homogeneously dispersed inorganic nanoparticles [5,6]. Possible solutions are the chemical modification of nanoparticle surface by various silanes to reduce surface energy or in-situ synthesis of nanoparticles inside the host polymer matrix [7,8].

Zinc oxide (ZnO), particularly its nanostructures, has recently attracted significant attention as a highly promising material for a broad range of applications [9,10,11]. ZnO is a frequently used semiconductor with high UV absorption, interesting electrical and optical properties, which strongly depend on the particle size and shape [12,13]. Besides, ZnO influences the thermal and mechanical properties as well as the chemical and physical stability of polymer matrices [14]. ZnO is known for its UV protecting capability [15,16,17], but its impact on the catalytic degradation of polymers has been less explored [18,19].

In the middle of the 1990s, it was discovered that ZnO also shows antibacterial activity against some bacterial strains. There are many studies on the preparation of ZnO/polymer nanocomposites with antibacterial activity [20,21]. For effective antibacterial activity, ZnO has to come into direct contact with the microorganisms. In the case of composites prepared with surface modified ZnO, antibacterial activity is compromised at the expense of better matrix stability.

Many publications reported on polyolefin/ZnO (nano)composites which were prepared by various processes. Here, we focus only on the composites prepared by melt processing (extrusion and injection molding). Some authors disclosed enhanced electrical properties (reduced resistivity for few orders of magnitude) [22,23,24] or UV stability [15,16,17] when large quantities of nano ZnO (30 wt% and more) were added to the polyolefin (PE or PP) matrices. Some of them reported on significantly improved antibacterial activity of PE or PP/ZnO nanocomposites [7,20,21,25]. Considering the mechanical properties of polyolefin/ZnO (nano)composites, some authors reported on significantly enhanced properties [15,26,27], while others stated only a minimal effect of nano ZnO addition [28,29,30]. Because interfacial interaction between ZnO and polyolefins is very weak [31], the surface of ZnO is frequently modified, mostly with silanes, in order to increase the compatibility between both composite constituents [27,30]. Nano ZnO can increase the degree of crystallinity of polyolefins [31,32], although many authors also reported its negligible effect [22,29]. Therefore, there are still many aspects of polyolefin/ZnO nanocomposites formation and properties, as well as potential applications that need to be cleared out.

Here, we report on the preparation of the composite materials of polyolefin matrices (HDPE and PP) and various types of commercially available nano ZnO (unmodified and surface modified with stearic acid, triethoxycaprylylsilane, and [3-methacryloxypropyl]trimethoxysilane) by deposition of nano ZnO on the surface of polyolefin granules and subsequent melt processing. Our goal was to introduce certain functionalities into the studied polymer matrices, such as UV absorption and antibacterial activity. Besides, the impact of ZnO addition on the composites’ mechanical and thermal properties, as well as UV-Vis stability, was studied.

## 2. Results and Discussion

### 2.1. Characterization of ZnO Nanofillers

**Figure 1:**
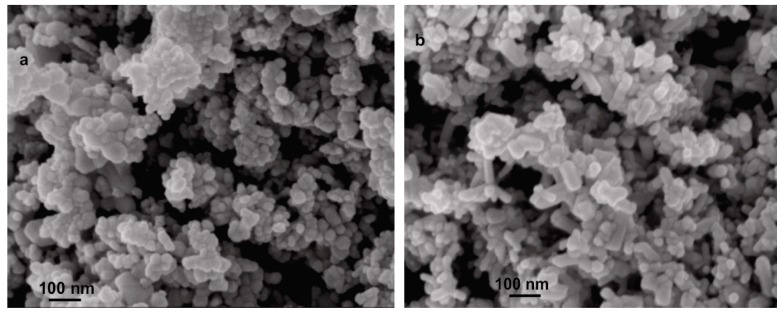
SEM micrographs of (**a**) Zinkoxyd aktiv; (**b**) Zano 20.

**Figure 2:**
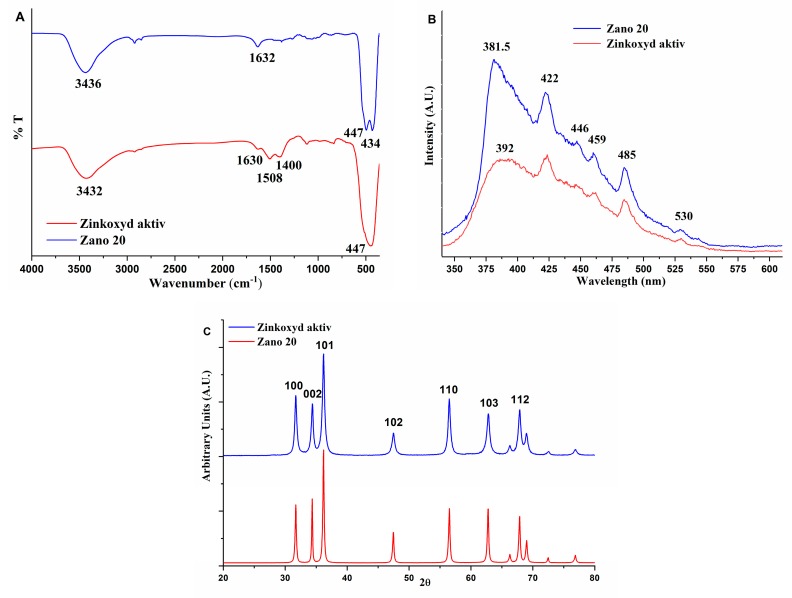
(**A**) FTIR spectra; (**B**) Photoluminescence spectra; (**C**) XRD diffractograms of commercial nano ZnO powders.

Samples of commercial zinc oxides (Zinkoxyd aktiv and Zano 20) were characterized before they were applied as the nanofillers. The specific surface area based on the BET method is 42.8 m^2^/g for Zinkoxyd aktiv and 25.1 m^2^/g for Zano 20, while the average pore dimensions are 16.2 nm and 7.6 nm, respectively. SEM micrographs show well-defined ZnO nanoparticles with particle sizes between 20 and 100 nm for both samples (Figure 1). Zano 20 contains a larger fraction of rodlike ZnO structures (Figure 1b), while the degree of particle agglomeration is higher for the Zinkoxyd aktiv. FTIR spectra of ZnO powders (Figure 2A) show characteristic strong and broad absorption bands between 420 and 450 cm^−1^ due to the two transverse optical stretching modes of ZnO [33]. In the FTIR spectrum of Zinkoxyd aktiv, consisting of ZnO nanoparticles with irregular spherical morphology, only broad absorption band with a maximum at 447 cm^−1^ is observed (Figure 2A), while the FTIR spectrum of Zano 20 shows broad band with two maxima, one at 447 cm^−1^ and the other one at 434 cm^−1^ (Figure 2A), which are characteristic of the rod-like ZnO morphology [34]. Besides, Zinkoxyd aktiv shows additional absorption bands at 1508 and 1400 cm^−1^, typical for the organic moieties most probably located on the surface of ZnO particles, indicating that Zinkoxyd aktiv is more organophillic than Zano 20. Photoluminescence spectra of both ZnO powders show the near band edge UV emission from 380 to 400 nm and numerous visible light emission peaks at 423, 448, 461, 485, and 529 nm [10,12] (Figure 2B). Differences between the samples in the visible light emission region are rather small, indicating small differences in the quantity and type of intrinsic defects on the surfaces of both ZnO samples [35]. A larger difference was observed in the near band edge peak, which is located at 381.5 nm and 392 nm for the Zano 20 and Zinkoxyd aktiv, respectively. The intensity of this peak is much higher for the Zano 20 and is most probably related to the rod-like ZnO morphology (thick rods) [36]. XRD diffractograms (Figure 2C) show diffraction maxima that are characteristic of the crystalline ZnO with hexagonalwurtzite structure (JCPDS card no. 01-079-0205) at 2θ values: 31.8, 34.5, 36.2, 47.6, 56.6, 62.9, 66.4, 67.9, 69.1, 72.6, and 76.9 [37]. Calculated average crystallite sizes are 16.4 nm for Zinkoxyd aktiv and 42.7 nm for Zano 20, indicating that the latter contains much larger crystallites than the former one. Based on these results, we conclude that the Zano 20 has larger crystallite size, lower specific surface area, and rod-like morphology, while the Zinkoxyd aktiv has irregular spherical morphology, larger specific surface area, and organic layer on the surface.

### 2.2. Composites of Nano ZnO with HDPE and PP Matrices; Unmodified ZnO and Surface-Modified ZnO with Stearic Acid

First, we studied the distribution of ZnO in polyolefin matrix by SEM microscopy using a backscattered electron detector. SEM micrographs in Figure S1A–D show the distribution of ZnO in the HDPE matrix at a concentration of nano ZnO of 1.0 wt%. The dimensions of ZnO are from 1 to 5 μm, indicating that ZnO is predominantly in the aggregated form. A comparison of the micrographs of the HDPE nanocomposites prepared by unmodified ZnO (Figure S1A,C) and with stearic acid modified ZnO (Figure S1B,D) indicates that stearic acid coating improves compatibility between the Zano 20 and HDPE, as indicated by a reduced number of large aggregates (Figure S1D).

**Figure 3:**
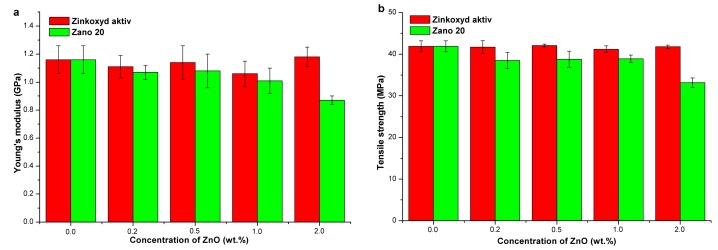
Mechanical properties of HDPE/ZnO composites as a function of nano ZnO concentration: (**a**) Young’s modulus and (**b**) Tensile strength.

Table S1 shows the mechanical properties of HDPE/ZnO composites (unmodified ZnO and with stearic acid (3.0 wt%) modified ZnO: Zinkoxyd aktiv and Zano 20) as a function of nano ZnO concentration. The results show that Zinkoxyd aktiv has no particular effect on the composites’ mechanical properties since they remain more or less unchanged with a slight downward trend. An exception is elongation at break, which shows a trend of slight increase (Table S1, Figure S2A). The HDPE nanocomposites with Zano 20 show reduction in the tensile strength and Young’s modulus and a more pronounced increase in elongation at break (Table S1 and Figure 3). It is obvious that the addition of nano ZnO slightly deteriorates the mechanical properties of the HDPE/ZnO nanocomposites. In particular, the decrease in tensile strength and Young’s modulus (by 21% and 25%, respectively) was pronounced when 2% by weight of Zano 20 was added (Figure 3 and Figure S2B). Obviously, such a high concentration of Zano 20 in HDPE is not beneficial. Surface modification of ZnO with stearic acid shows slightly enhanced compatibility between ZnO and HDPE (Figure S1), but no improvement of composites’ mechanical properties was observed (Table S1).

Study of thermal properties (melting temperature and melting enthalpy) of HDPE/ZnO composites (unmodified Zinkoxyd aktiv and Zano 20) as a function of nano ZnO concentration shows that the addition of nano ZnO only slightly affects the melting temperature, ΔH_m_, and crystallinity degree of HDPE matrix (Table S1, Figure S3). Only small changes in the degree of HDPE crystallinity, together with a rather high degree of ZnO aggregation (Figure S1), are explanations for a minimal impact of added nano ZnO on the nanocomposites’ mechanical properties. Due to a high degree of ZnO aggregation, the interface surface between ZnO and HDPE is rather small. On the other hand, a major mechanism influencing the nanocomposite mechanical properties is that inorganic nanostructures act as the crystallization nuclei, resulting in a higher crystallinity of the polymer matrix and thus in improved mechanical properties. Since, in our case, the crystallinity degree is not affected by ZnO, this explains only the small changes in observed mechanical properties (Table S1). In literature, some authors reported on significant increase in crystallinity by the addition of nano ZnO [31], while others observed no changes [22]. Consequently, some authors reported on improved composite mechanical properties [7,15] and others did not [2,28]. On the other hand, TGA results reveal improved thermal stability of the HDPE/ZnO composites compared to the neat HDPE (Figure S4).

SEM micrographs in Figure S5A–D show the distribution of ZnO in the PP matrix at 1 wt% concentration of nano ZnO. The ZnO particles are predominantly present in PP matrix as the aggregates with sizes varying from 1 to 5 μm. A comparison of micrographs (unmodified ZnO—Figure S5A,C and ZnO modified with stearic acid, Figure S5B,D) shows that stearic acid primarily improves the compatibility of Zano 20 with PP matrix, as indicated by the smaller number of large aggregates (Figure S5D).

**Figure 4:**
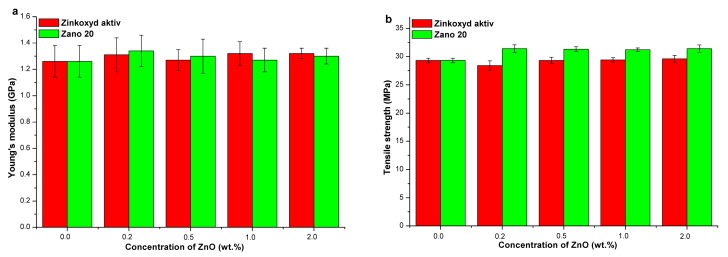
Mechanical properties of PP/ZnO composites as a function of nano ZnO concentration: (**a**) Young’s modulus and (**b**) Tensile strength.

The results of mechanical properties testing of PP/ZnO composites (unmodified ZnO and modified with stearic acid: 3.0 wt%) prepared by Zinkoxyd aktiv or Zano 20 nanofillers as a function of nanoparticle concentration are presented in Table S2 and Figure 4. Zinkoxyd aktiv does not have a significant influence on the composites’ mechanical properties at concentrations up to 2.0 wt% (Table S2, Figure S6A). On the other hand, Zano 20 shows a slightly more pronounced effect as indicated by increased tensile strength by 7.3% and Young’s modulus by 6.3% (Table S2, Figure S6B). Additionally, unmodified ZnO slightly increases Young’s modulus, while this was not observed for the ZnO modified with stearic acid, which is attributed to the plasticizing effect of stearic acid.

Table S2 also summarizes the thermal properties (melting temperature and melting enthalpy) of PP/ZnO composites (Zinkoxyd aktiv and Zano 20) depending on the ZnO concentration. Nano ZnO has no significant effect on the melting temperature, ΔH_m_, and degree of PP crystallinity (Table S2, Figures S7 and S8). The literature dealing with the effect of ZnO on the PP crystallinity are dubious since some authors reported on increased PP crystallinity [26,32], while others observed no changes, or even the opposite effect [29]. Similar to the case of HDPE/ZnO composites, only minimal changes in the degree of PP matrix crystallinity and a rather high degree of ZnO aggregation (Figure 1) are the explanations for the small effect of added nano ZnO on the mechanical properties of PP nanocomposites (Table S2). ZnO addition to the PP can cause various effects on its crystallinity, depending on the composite preparation process; consequently, different effects on the mechanical properties are reported. Some authors reported on enhancement of mechanical properties [22,26,27], while others reported on negligible effect of added nano ZnO [29,38].

Many research groups also studied the effect of ZnO on the UV stability of polymer matrices. Literature reports enhanced polymer UV-stability since ZnO is an excellent UV absorber, however, the absorbed energy can be transferred to the polymer chains, causing their scission. Upon UV light exposure, ZnO is excited, leading to the formation of oxygen-active (hydroperoxide) species in the presence of OH groups on the surface, which can cause scission of polymer chains [17,39]. The first change caused by the UV light is the color or gloss change [40]. Therefore, the color change of polyolefin/ZnO nanocomposites was measured as a function of the exposure time of the composite to the artificial sun light.

Figures S9 and S10 show changes in the color (ΔE) of HDPE/ZnO composites (Zinkoxyd aktiv—Figure S9 and Zano 20—Figure S10), depending on the exposure time to the artificial sunlight. ΔE up to 7 weeks does not exceed the value of 4, meaning that with the naked eye, the color change is barely detectable. After ten weeks of exposure, ΔE reached the values from 8 to 15, meaning that color change was easily detected by naked eye. The change in color also depends on the nano ZnO concentration, since the largest color changes were observed at the highest nano ZnO concentration (2.0 wt%). Surface modification of nano ZnO with stearic acid reduces color changes (Figures S9 and S10). A comparison of both ZnO nanoparticles revealed that Zano 20 has a more pronounced effect on the color change of HDPE/ZnO composites than Zinkoxyd aktiv, which is in line with the presence of organic layer on the surface of Zinkoxyd aktiv. Other authors reported on similar effects of ZnO and TiO_2_ particles on the UV stability of PE [19,39].

Figure S11 shows a color change of the PP/ZnO composites as a function of exposure time to the artificial sunlight. ΔE does not exceed 4, meaning only a moderate color change over ten weeks of exposure, which is equal to two years in real sunlight. The color change depends on the nano ZnO concentration, since the largest change was observed at 2.0 wt% of added ZnO. Surface modification of nano ZnO with stearic acid reduces the effect of nano ZnO, similar to the case of HDPEP/ZnO composites (Figure S11). Compared to HDPE, the PP matrix shows a higher stability to the artificial sunlight (Figure S9), indicating that a lifetime of PP/ZnO composites significantly exceeds two years even at 2.0 wt% of nano ZnO (Zinkoxyd aktiv).

HDPE/ZnO and PP/ZnO composites aged under artificial sunlight were studied by FTIR-ATR spectroscopy to perceive possible chemical processes and changes in chemical composition that occurred in the material (Figures S12 and S13). The FTIR spectra in Figure S12A–C show only minor differences compared to those of neat HDPE treated in the same way, leading to a conclusion that observed color changes (Figures S9 and S10) are not in correlation with the chemical changes (degradation) of the studied HDPE/ZnO composite samples. A comparison of FTIR spectra of PP/ZnO composites without and with 1.0 wt% of ZnO exposed to sunlight (Figure S13A–C) show the appearance of a new absorption band at 1725 cm^−1^, corresponding to the formation of carbonyl groups. We studied its intensity as a function of composite exposure time (Figure S14). Results reveal increasing intensity of the carbonyl absorption band with time, but differences between the pure PP and PP/ZnO composites (unmodified and modified) are negligible, so no correlation can be established with the ZnO concentration in the PP matrix. We concluded that carbonyl absorption band is not related to the presence of ZnO [40].

### 2.3. Composites of Nano ZnO with HDPE and PP Matrix—ZnO Surface Modified with Silanes

HDPE and PP composites were prepared also with commercially available nano ZnO powders, the surface of which was modified with silanes (Zano 20 Plus: 3.9 wt% of caprylyl silane and Zano 20 Plus 3: 1.0 wt% of methacrylic silane). SEM micrographs in Figure S15 show distributions of silanized ZnO particles in composites based on HDPE and PP matrices. In comparison with composites prepared with unmodified ZnO and ZnO modified with stearic acid (Figures S1 and S5), the micrographs in Figure S15 show the presence of significantly smaller ZnO aggregates (size below 2 µm), confirming that silanization of ZnO considerably improves compatibility between the polyolefin matrix and nano ZnO.

**Figure 5:**
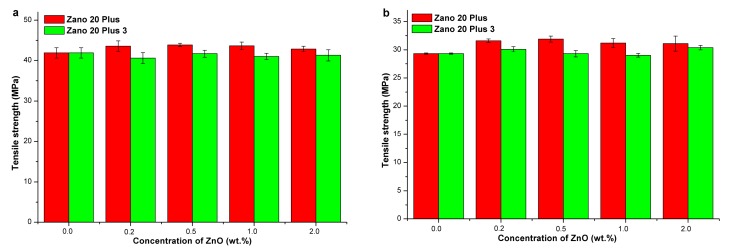
Tensile strength of polyolefin/ZnO composites as a function of silanized nano ZnO concentration (Zano 20 Plus): (**a**) HDPE and (**b**) PP matrix.

Table S3 summarizes the mechanical properties of HDPE/silanized ZnO. When Zano 20 Plus nanofiller was used, a slight increase in tensile strength (by up to 4.7%—Figure 5a, Figure S16A) and elongation at break was observed, while Young’s modulus is somewhat reduced. In the case of Zano 20 Plus 3, only elongation at break slightly increased, while tensile strength and Young’s modulus were slightly reduced. The reduction of Young’s modulus is attributed to the presence of silane, acting as the plasticizer. Overall, silanized ZnO has only a minor effect on the mechanical properties of ZnO composites with HDPE matrix as indicated by enhancement of Young’s modulus and tensile strength by 2.0% and 4.7%, respectively. These results are in accordance with the composites’ thermal properties, showing no relationship with ZnO concentration (Table S3). Some authors, on the other hand, reported on the enhancement of composites’ mechanical properties when ZnO was modified with the silane coupling agents [7,31].

Table S4 summarizes the mechanical properties of PP/silanized ZnO composites. When Zano 20 Plus was used, the tensile strength (by up to 8.7%—Figure 5b, Figure S16B) and elongation at break increased, while Young’s modulus slightly decreased. In the case of Zano 20 Plus 3, only elongation at break increases, while tensile strength and Young’s modulus are more or less unaffected. Overall, the silanized ZnO has a rather small influence on the mechanical properties of composites based on the PP matrix since the maximal enhancement of tensile strength is 8.7%. Young’s modulus is reduced in most cases, which is attributed to the plasticizing effect of silane. The thermal properties of PP/silanized ZnO composites showed a substantial increase (14.3%) in melting enthalpy when Zano 20 Plus was used (Table S4, Figure S17), while composites with Zano 20 Plus 3 showed no significant changes. These observations are in accordance with an increase in tensile strength (Table S4) when Zano 20 Plus nanofiller was used, confirming that an increase in crystallinity degree is a predominant mechanism of mechanical properties reinforcement. These results are in contrast to those results, reporting on negligible changes of mechanical properties when silane coupling agents were applied [27,30], but in line with those reporting on enhancement of composite mechanical properties when nano ZnO surface modified with methacrylic silane was applied [32].

Figures S18 and S19 show color changes of HDPE/ZnO and PP/ZnO composites (Zano 20 Plus and Zano 20 Plus 3), depending on the composite exposure time to artificial sunlight and ZnO concentration. The change in color strongly depends on the ZnO concentration, since it is the greatest at 2.0 wt% (Figures S18 and S19). Moreover, a thicker layer of silane (Zano 20 Plus—3.9 wt% of caprylyl silane) slightly reduces color change as compared to the thinner layer (Zano 20 Plus 3–1.0 wt% methacrylic silane). A comparison of color changes shows that HDPE is significantly more light-sensitive than PP, since the latter shows only moderate changes in the color metrics after 10 weeks of exposure time; ΔE is below 4 for PP and between 6 and 11 for HDPE (Figures S18 and S19).

### 2.4. Antibacterial Properties of Polyolefin Composites with ZnO Nanoparticles

ZnO is well known as a highly efficient antibacterial agent. The exact mechanism of ZnO antibacterial activity is still not completely elucidated. There are many possible physical and chemical mechanisms of the ZnO interaction with the bacterial cells: (a) generation of reactive oxygenated species (ROS = O_2_^−•^, HO_2_^•^ and HO^•^) under UV or visible light illumination; (b) release of Zn^2+^ ions due to partial dissolution of ZnO; (c) generation of H_2_O_2_ by photoinduction; (d) disruption of plasma membrane due to interaction with ZnO; (e) internalization (penetration) of ZnO nanoparticles into the bacterial cell; (f) mechanical damage of the cell membrane [41]. The first three interaction mechanisms are of a chemical nature, while the other three are physical. The real effect of ZnO on the bacterial cells is most probably a combination of these mechanisms.

**Figure 6:**
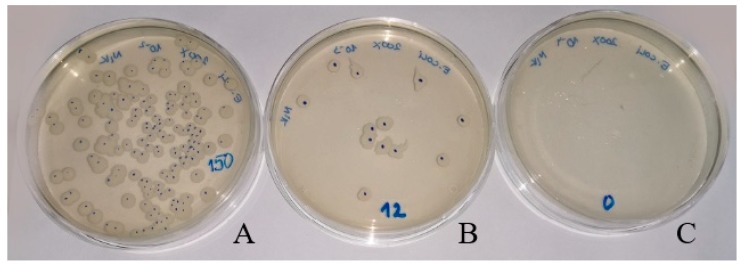
Determination of *E. coli* cell number (CFU/mL): (**A**) Starting inoculum concentration of *E. coli*; (**B**,**C**) *E. coli* count on two parallel test samples of HDPE/unmodified ZnO composite after 24 h.

The measurements of antibacterial activity were carried out on the selected samples (Table 1) of ZnO composites with polyolefin matrices (Table 2, Table 3). We selected the samples containing 2.0 wt% of ZnO either Zinkoxyd aktiv or Zano 20, and ZnO of different surface modification. According to ISO 22196: 2007 standard, the log of reduction must be equal to 2 or higher than 2 so that certain material can be accepted as antibacterial [42]. The results show that all composites have excellent antibacterial activity against *S. aureus* (gram-positive bacteria) (Table 3), while the results on antibacterial activity against *E. coli* (gram-negative bacteria) vary significantly (Table 2) [41]. It is known that ZnO shows a more intense effect on the gram-positive bacteria such as *S. aureus* or *Bacillus subtilis* than on the gram-negative bacteria such as *E. coli* or *Aerobacter aerogenes* [25,43,44,45]. This can be attributed to their structural and compositional differences. Namely, gram-negative bacteria have an additional outer plasma membrane that consists of a thick lipopolysaccharide layer. The overall thickness of the membrane is larger in the gram-negative bacteria than in the gram-positive ones. These structural differences are the most probable reason for higher resistance of gram-negative bacteria towards ZnO [43,44,45]. Photographs of bacterial colonies on agar (Figure 6), taken after 24 h of contact with HDPE/ZnO composites containing 2.0 wt% of unmodified nano ZnO (Figure 6B,C), present a significant reduction in the number of *E. coli* bacterial colonies.

Samples of surface unmodified ZnO show good or excellent activities, while those of surface modified ZnO are poor or even bad (Table 2). Comparing both ZnO nanofillers, Zano 20 shows higher antibacterial activity than Zinkoxyd aktiv, but the difference is mainly in the case of *E. coli*. Based on these results, we conclude that for achieving sufficient antibacterial activities towards both types of bacteria, the application of commercial unmodified Zano 20 ZnO in concentration of 2.0 wt% is recommended, although unmodified Zinkoxyd aktiv also show sufficient antibacterial activity with polyolefin matrices. The observed differences between *E. coli* and *S. aureus* in the antibacterial effect of polyolefin/ZnO composites originate from different membrane structure of gram-negative and gram-positive bacteria. Concerning the possible interaction mechanisms between ZnO and bacteria, we assume that chemical mechanisms do not require a physical contact between the ZnO particle and bacterial membrane since ROS, Zn^2+^ ions, and H_2_O_2_ can migrate from the surface of ZnO to the surface of bacteria. We conclude that the membrane structure of *S. aureus* allows the penetration of antibacterial active chemical compounds into the cell, thus explaining why, in this case, surface modified ZnO is also effective (Table 2). On the other side, the cell of *E. coli* is most probably damaged or destroyed only by the physical processes, requiring physical contact with ZnO, which thus explains why, in this case, only the unmodified ZnO is effective against this type of bacteria (Table 2).

## 3. Materials and Methods

### 3.1. Materials

Commercially available TIPELIN BA 550-13 HDPE and TIPPLEN K-499 PP granules, and ZnO nanopowders: Zinkoxyd aktiv, Lanxsess, Germany and Zano 20, Umicore, Belgium. In addition, commercial silane modified Nano ZnO nanofillers were also applied: Zano 20 Plus, Zano 20 Plus 3, Umicore, Belgija. The Zano 20 Plus was surface modified with 3.9 wt% of triethoxycaprylylsilane caprylylsilane, while the Zano 20 Plus 3 was modified with 1 wt% of [3-methacryloxypropyl]trimethoxysilane methacrylic silane. *Escherichia coli* strain (DSM 498) and *Staphilococcus aureus* strain (DSM 346) were supplied by DSMZ-Deutsche Sammlung von Mikroorganismen und Zellkulturen GmbH, Germany.

### 3.2. Preparation of Polyolefin/ZnO Composites

ZnO nanopowders were applied to the surface of the PE or PP granulate. For this purpose, nano ZnO was first suspended in ethanol and subsequently the PE or PP granulate was added and sonicated. Finally, ethanol was evaporated on the rotary evaporator. The prepared granulates were extruded at 160 °C (PE) or 180 °C (PP) for 10 min at 50 rpm with Haake MiniLab extruder (Thermo Fischer Scientific, Karlsruhe, Germany). The mixture was added to the extruder in two portions of 3 g. The extruded melt was ejected from the extruder at 100 rpm and it was captured in a heated container (170 °C), which was further placed into a Haake Mini Jet molding machine (Thermo Fischer Scientific, Karlsruhe, Germany) to prepare the testing specimens by injection into a suitable mold heated to 70 °C at the pressure of 750 bar and time of 10 s, as well as the post pressure of 250 bar and time of 10 s.

### 3.3. Characterization

Mechanical properties were measured according to ISO 527 standard on the Shimadzu AGS-GX (Shimadzu, Kyoto, Japan) plus a dynamometer with an initial spacing of 58 mm and the stretching speed of 2 mm/min and 200 mm/min.

UV-vis resistance of composites was determined by exposing the samples to artificial sunlight in the Suntest chamber for certain time periods. Color change was measured as a function of time by using an i1 Pro (X-Rite, Grand Rapids, MI, USA) spectrometer, measuring the spectra of reflectivity in a spectral range of 380–730 nm.

Fourier-transform infrared spectroscopy (FTIR) spectra were recorded in a transmittance mode on a FTIR spectrometer Spectrum One (Perkin-Elmer, Waltham, MA, USA) in a spectral range between 360 cm^−1^ and 4000 cm^−1^, and a spectral resolution of 4 cm^−1^ using KBr tablets (ZnO powders) or in an ATR mode in a spectral range from 650 to 4000 cm^−1^ with a 4 cm^−1^ spectral resolution (polyolefin/ZnO composites).

Photoluminescence spectra of ZnO powders were recorded on a Perkin Elmer LS-55 spectrometer (Perkin-Elmer, Waltham, MA, USA) in a range from 330 nm to 620 nm using an excitation wavelength of 325 nm.

SEM micrographs of composites were taken on a Zeiss Supra 35 VP field emission electron microscope (Zeiss, Oberkochen, Germany) at a 15 kV acceleration voltage using a back-scattered electron detector at a working distance of 8 mm.

Thermal properties of polyolefin matrices and composites were determined by DSC calorimetry on DSC-1 (Mettler Toledo, Greifensee, Switzerland) in a temperature range from 25 °C to 200 °C at a heating rate of 10 K/min.

Thermal stability of composite samples was determined by thermogravimetric analysis (TGA) in oxygen atmosphere using a TGA-1 (Mettler Toledo, Greifensee, Switzerland) instrument. The measurements were performed in a temperature range from 30 °C to 800 °C at a heating rate of 10 K/min.

Antibacterial activity of ZnO/polyolefin composites was determined according to ISO 22196: 2007 standard.

Crystalline fractions of the ZnO powders were characterized by a wide-angle X-ray diffraction (XRD) on an XPert Pro diffractometer (PANalytical, Almelo, Netherlands) with Cu anode as an X-ray source. X-ray diffractograms were measured at 25 °C in the 2θ range from 5° to 80° with a step of 0.033° and step time of 100 s. Crystallite sizes were calculated using the Scherrer formula [10] and Si wafer was used to determine the experimental peak broadening.

Nitrogen sorption measurements were performed on a manometric gas sorption analyzer (Micromeritics Instrument Co., Norcross, GA, USA) at −196 °C in the range of relative pressure values from 10^−6^ to 1. As-prepared samples were degassed at 140 °C for 16 h prior to the measurements. The specific surface areas were determined by BET method based on the obtained sorption isotherms.

## 4. Conclusions

SEM microscopy revealed agglomerated nano ZnO with sizes from 1 to 5 μm in polyolefin matrices. Results of tensile testing showed that both nano ZnO do not enhance the mechanical properties of HDPE composites, while PP composites show slight enhancement (Young’s module by 6% and tensile strength by 7%) when Zano 20 was used as a nanofiller. Measurements of thermal properties revealed only a small effect of nano ZnO on the degree of crystallinity of these composites, which is in accordance with the unchanged mechanical properties. Surface modification of ZnO with stearic acid increases its compatibility with polyolefin matrices, which, however, does not result in improved composite mechanical properties. Color change of composite materials was studied in dependence of ZnO concentration, exposure time to artificial sunlight, and ZnO type. Higher nano ZnO concentrations cause more obvious color changes of HDPE/ZnO composites, while surface modification of ZnO with stearic acid partially reduces this effect. Nevertheless, the PP matrix shows significantly smaller changes (barely visible with naked eye—ΔE is below 4) than the HDPE matrix (ΔE is between 8 and 15) after 10 weeks of exposure of composites to artificial sunlight. As revealed by TGA, added nano ZnO (both ZnO types—2.0 wt%) also increased the thermal stability of HDPE while no changes were observed for PP matrix.

Silanization of nano ZnO significantly improved compatibility of ZnO with polyolefin matrices as indicated by smaller ZnO agglomerates (1–2 μm). Despite better ZnO distribution in polyolefin matrices, it has only a minor effect on the composites’ mechanical properties. Nevertheless, clear positive trends were observed with silanized nano ZnO as indicated by 5–9% enhanced tensile strength when caprylyl silane (3.9 wt%) was applied as the ZnO surface modifying agent in the PP matrix. This was accompanied by 14.3% increase in the PP melting enthalpy, confirming that increased PP crystallinity is responsible for the enhanced composite mechanical properties. Young’s modulus is reduced, which is attributed to the plasticizing effect of silane. Silanization also slightly reduces color changes of polyolefin/ZnO composites. Concerning improvement of mechanical properties in general, the use of nano ZnO powders as the reinforcing agents in polyolefins is economically not justified.

The composites with unmodified and surface modified ZnO are highly active against *S. aureus*, while in the case of *E. coli*, only the composites with unmodified ZnO show sufficient antibacterial activity. This difference is attributed to differences in membrane structure between the gram-positive (*S. aureus*) and gram-negative (*E. coli*) bacteria. To achieve sufficient antibacterial activity of polyolefin composites towards both bacteria types, the application of unmodified commercial ZnO (Zano 20) in the concentration of 2.0 wt% is recommended. Incorporation of ZnO into the polyolefin matrices is considered to be a promising way to improve material antibacterial properties for clinical application or applications in the food industry.

## Figures and Tables

**Table 1:** Data on samples of HDPE or PP/ZnO composites selected for antibacterial activity testing (the concentration of nano ZnO is 2.0 wt%). Zinkox-aktiv = Zinkoxyd aktiv.

Designation of Samples	Polymer Matrix	Type of Nano ZnO	Type of Modification	Amount of Modifier (wt%)
PEZnO-1	HDPE	Zinkox-aktiv	-	-
PEZnO-2	HDPE	Zano 20	-	-
PEZnO-3	HDPE	Zano 20	Stearic acid	3.0
PEZnO-4	HDPE	Zano 20 Plus	Caprylyl silane	3.9
PPZnO-1	PP	Zinkox-aktiv	-	-
PPZnO-2	PP	Zano 20	-	-
PPZnO-3	PP	Zinkox-aktiv	Stearic acid	3.0

**Table 2:** Determined antibacterial activities and adequate activities scores of HDPE/ZnO and PP/ZnO composites against *E. coli*. Zinkox-aktiv = Zinkoxyd aktiv.

Designation of Samples	Type of Nano ZnO	Ref. (t = 0)	Ref. (t = 24 h)	Sample (t = 24 h)	U_0_	U_t_	A_t_ (log_10_ (CFU/mL))	R	Score
		[CFU/mL]	[CFU/mL]	[CFU/mL]					
PEZnO-1	Zinkox-aktiv	3.9 × 10^3^	3.6 × 10^3^	3.1 × 10^1^	3.39	3.35	1.29	2.06	Good
PEZnO-2	Zano 20	3.9 × 10^3^	3.6 × 10^3^	1	3.39	3.35	−0.20	3.55	Excellent
PEZnO-3	Zano 20	3.9 × 10^3^	3.6 × 10^3^	4.2 × 10^2^	3.39	3.35	2.42	0.92	Poor
PEZnO-4	Zano 20 Plus	3.9 × 10^3^	3.6 × 10^3^	1.3 × 10^3^	3.39	3.35	2.91	0.44	Bad
PPZnO-1	Zinkox-aktiv	3.8 × 10^3^	3.6 × 10^3^	4.4 × 10^2^	3.38	3.35	2.44	0.91	Poor
PPZnO-2	Zano 20	3.8 × 10^3^	3.6 × 10^3^	3	3.38	3.35	0.27	3.08	Excellent
PPZnO-3	Zinkox-aktiv	3.8 × 10^3^	3.6 × 10^3^	5.9 × 10^2^	3.38	3.35	2.56	0.78	Poor

**Table 3:** Determined antibacterial activities and adequate activities scores of HDPE/ZnO and PP/ZnO composites against *S. aureus*. Zinkox-aktiv = Zinkoxyd aktiv.

Designation of Samples	Type of Nano ZnO	Ref. (t = 0)	Ref. (t = 24 h)	Sample (t = 24 h)	U_0_	U_t_	A_t_ (log_10_(CFU/mL))	R	Score
		[CFU/mL]	[CFU/mL]	[CFU/mL]					
PEZnO-1	Zinkox-aktiv	1.1 × 10^4^	1.2 × 10^4^	1.5	3.83	3.89	−0.03	3.91	Excellent
PEZnO-2	Zano 20	1.1 × 10^4^	1.2 × 10^4^	0.5	3.83	3.89	−0.50	4.39	Excellent
PEZnO-3	Zano 20	1.1 × 10^4^	1.2 × 10^4^	1	3.83	3.89	−0.03	4.09	Excellent
PEZnO-4	Zano 20 Plus	1.1 × 10^4^	1.2 × 10^4^	2	3.83	3.89	0.10	3.79	Excellent
PPZnO-1	Zinkox-aktiv	8.7 × 10^3^	8.4 × 10^3^	1	3.73	3.72	−0.20	3.92	Excellent
PPZnO-2	Zano 20	8.7 × 10^3^	8.4 × 10^3^	0.5	3.73	3.72	−0.50	4.22	Excellent
PPZnO-3	Zinkox-aktiv	8.7 × 10^3^	8.4 × 10^3^	1.5	3.73	3.72	−0.03	3.75	Excellent

## Supplementary Materials

The following Figures and Tables are available online, Figure S1: SEM micrographs (magnification 1000×, backscattered electron detector) of HDPE/ZnO composites (Zinkoxyd aktiv – 1.0 wt%): (A) unmodified nano ZnO - Zinkoxyd aktiv; (B) nano ZnO - Zinkoxyd aktiv modified with stearic acid; (C) unmodified nano ZnO - Zano 20; (D) nano ZnO-Zano 20 modified with stearic acid, Table S1: Mechanical and thermal properties of HDPE/ZnO (Zinkoxyd aktiv or Zano 20) composites as the functions of nano ZnO concentration and surface modification of ZnO with stearic acid. The polymer matrix is polyethylene HDPE - H-3581, the addition of stearic acid is 3% by weight, Znoxaktiv is Zinkoxyd-aktiv, Figure S2: Stress-strain curves of HDPE/ZnO composites as a function of ZnO concentration: 0 wt% - black; 0.5 wt% - green; 2.0 wt% blue: (A) Zinkoxyd aktiv; (B) Zano 20, Figure S3: DSC curves of HDPE/ZnO composites as a function of ZnO concentration, Figure S4: TGA curves of HDPE/ZnO composites as a function of ZnO concentration, Figure S5: SEM micrographs (magnification 1000×, backscattered electron detector) of PP/ZnO composites (Zinkoxyd aktiv – 1.0 wt%): (A) unmodified nano ZnO - Zinkoxyd aktiv; (B) nano ZnO - Zinkoxyd aktiv modified with stearic acid; (C) unmodified nano ZnO - Zano 20; (D) nano ZnO-Zano 20 modified with stearic acid, Table S2: Mechanical and thermal properties of composites with PP matrix and ZnO - Zinkoxyd aktiv or Zano 20 (unmodified ZnO and surface modified with stearic acid) depending on the nano ZnO concentration. The polymer matrix is polypropylene PP - K499, the addition of stearic acid is 3.0 wt%, Znox-aktiv is Zinkoxyd aktiv, Figure S6: Stress-strain curves of PP/ZnO composites as a function of ZnO concentration: 0 wt% - black; 0.5 wt% - green; 2.0 wt% blue: (A) Zinkoxyd aktiv; (B) Zano 20, Figure S7: DSC curves of PP/ZnO composites as a function of ZnO concentration, Figure S8: TGA curves of PP/ZnO composites as a function of ZnO concentration, Figure S9: Changes in color of HDPE/ZnO composites (Zinkoxyd aktiv) as the functions of exposure time to artificial sunlight, nano ZnO concentration, and ZnO surface modification with stearic acid, Figure S10: Changes in color of HDPE/ZnO composites (Zano 20) as the functions of exposure time to artificial sunlight, nano ZnO concentration, and ZnO surface modification with stearic acid, Figure S11: Changes in color of PP/ZnO composites (Zinkoxyd aktiv) as the functions of exposure time to the artificial sunlight and ZnO concentration, Figure S12: FTIR spectra of HDPE/ZnO composites prior to exposure to artificial light and after ten weeks of exposure: (A) pure HDPE; (B) HDPE with 1.0 wt% of ZnO (Zinkoxyd-aktiv); (C) HDPE with 1.0 wt% of ZnO (Zano 20), Figure S13: FTIR spectra of PP/ZnO composites prior to exposure to artificial light and after ten weeks of exposure: (A) pure PP; (B) PP with 1.0 wt% of ZnO (Zinkoxyd-aktiv); (C) PP with 1.0 wt% of ZnO (Zano 20), Figure S14: Intensity of the absorption band at 1725 cm^−1^ in PP/ZnO composites as a function of exposure time to the artificial sunlight, Figure S15: SEM micrographs (magnification 1000×, backscattered electron detector) of HDPE/ZnO composites with 1.0 wt% of silane modified ZnO: (A) Zano 20 Plus; (B) Zano 20 Plus 3, and PP/ZnO composites: (C) Zano 20 Plus; (D) Zano 20 Plus 3, Table S3: Mechanical and thermal properties of composites of HDPE matrix and silanized nano ZnO (Zano 20 Plus - 3.9 wt% of caprylyl silane and Zano 20 Plus 3 – 1.0 wt% of methacrylic silane), Table S4: Mechanical and thermal properties of composites with PP matrix and silanized nano ZnO (Zano 20 Plus – 3.9 wt% of caprylyl silane and Zano 20 Plus 3 – 1.0 wt% of methacrylic silane), Figure S16: Stress-strain curves of polyolefin/ZnO composites as a function of silanized ZnO concentration (Zano 20 Plus): 0 wt% - black; 0.5 wt% - green; 2.0 wt% blue. (A) HDPE and (B) PP, Figure S17: DSC curves of PP/ZnO composites as a function of silanized ZnO concentration (Zano 20 Plus), Figure S18: Change in color of HDPE/ZnO composites (Zano 20 Plus and Zano 20 Plus 3) depending on the exposure time to artificial sunlight and nano ZnO concentration as well as the type of surface modification, Figure S19: Change in color of PP/ZnO composites (Zano 20 Plus and Zano 20 Plus 3) as the functions of the exposure time to artificial sunlight and nano ZnO concentration as well as the type of surface modification.
