# Supplementary material for: Polyolefin/ZnO Composites Prepared by Melt Processing
Source: Molecules. 2019 Jul 2;24(13):2432. doi: 10.3390/molecules24132432 (PMC6651218; doi:10.3390/molecules24132432)
Supplement: Supplementary file 1 [file molecules-24-02432-s001.pdf]

Supplementary Materials

Polyolefin/ZnO Composites Prepared by Melt Processing

Alojz Anžlovar [1,*], Mateja Primožič [2], Iztok Švab [3], Maja Leitgeb [2], Željko Knez [2] and Ema Žagar [1,*]

[1] Department of Polymer Chemistry and Technology, National Institute of Chemistry, Hajdrihova 19, SI-1000 Ljubljana, Slovenia
[2] University of Maribor, Faculty of Chemistry and Chemical Engineering, Smetanova ulica 17, SI-2000 Maribor, Slovenia; mateja.primozic@um.si (M.P.); maja.leitgeb@um.si (M.L.); zeljko.knez@um.si (Ž.K.)
[3] ISOKON d.o.o., Industrijska cesta 16, SI-3210 Slovenske Konjice, Slovenia; iztok.svab@isokon.si
* Correspondence: alojz.anzlovar@ki.si (A.A.); ema.zagar@ki.si (E.Ž.); Tel.: +386-1-4760-204 (A.A.); +386-1-4760-203 (E.Ž.); Fax.: +386-1-4760-300 (A.A. & E.Ž.)

Academic Editor: Marinella Striccoli, Roberto Comparelli and Annamaria Panniello

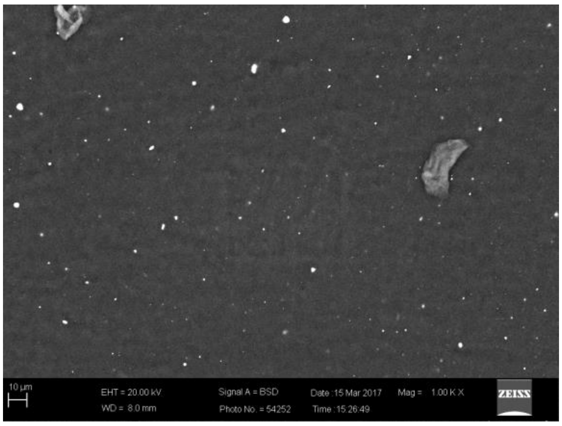

(A)

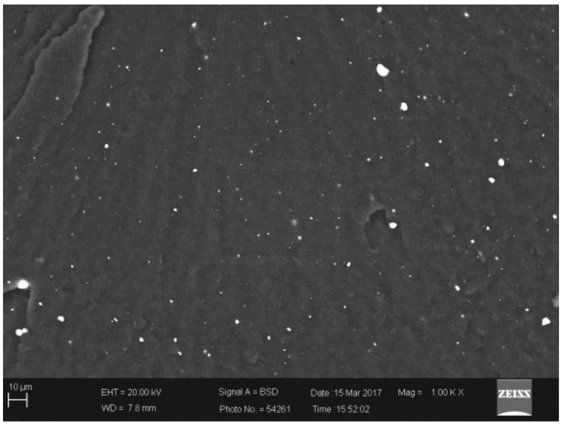

(B)

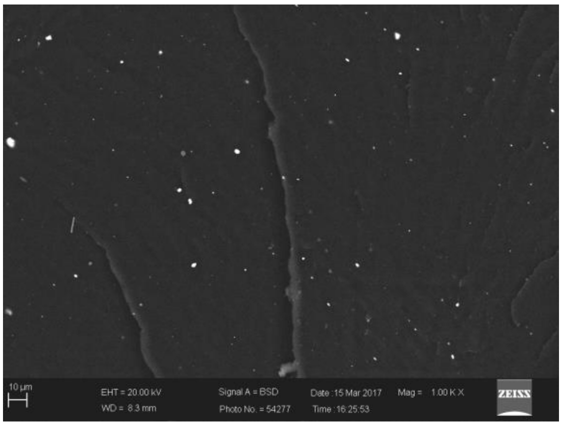

(C)

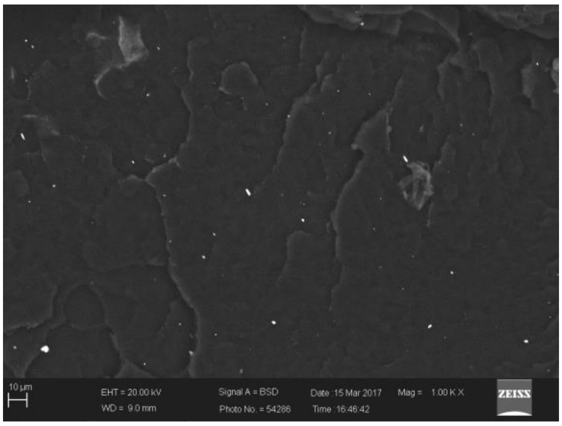

(D)

Figure S1. SEM micrographs (magnification 1000×, backscattered electron detector) of HDPE/ZnO composites (Zinkoxyd aktiv – 1.0 wt%): A) unmodified nano ZnO - Zinkoxyd aktiv; B) nano ZnO - Zinkoxyd aktiv modified with stearic acid; C) unmodified nano ZnO - Zano 20; D) nano ZnO-Zano 20 modified with stearic acid.

Table S1. Mechanical and thermal properties of HDPE/ZnO (Zinkoxyd aktiv or Zano 20) composites as the functions of nano ZnO concentration and surface modification of ZnO with stearic acid. The polymer matrix is polyethylene HDPE - H-3581, the addition of stearic acid is 3% by weight, Znox-aktiv is Zinkoxyd-aktiv.

Type of ZnO	Conc. of ZnO wt%	Type of modification	Young's modulus GPa	Tensile strength MPa	Strain at break %	Melting temp. °C	ΔH_m J g^{-1}
HDPE	0	-	1.16 ± 0.10	41.9 ± 1.31	28.3 ± 3.07	136.8	197.2
Znox-aktiv	0.2	-	1.11 ± 0.08	41.7 ± 1.52	31.6 ± 4.52	138.6	194.0
Znox-aktiv	0.5	-	1.14 ± 0.12	42.1 ± 0.33	30.3 ± 1.60	138.2	195.8
Znox-aktiv	1.0	-	1.06 ± 0.09	41.2 ± 0.82	29.7 ± 3.53	136.1	198.1
Znox-aktiv	2.0	-	1.18 ± 0.07	41.8 ± 0.40	29.0 ± 2.78	135.9	197.4
Znox-aktiv	0.2	Stearic acid	1.00 ± 0.10	40.0 ± 3.51	31.3 ± 3.85	134.6	197.9
Znox-aktiv	0.5	Stearic acid	1.13 ± 0.07	40.4 ± 2.07	30.7 ± 3.21	136.4	197.2
Znox-aktiv	1.0	Stearic acid	1.21 ± 0.09	40.4 ± 2.73	30.4 ± 2.08	137.5	196.7
Znox-aktiv	2.0	Stearic acid	1.20 ± 0.08	40.9 ± 2.77	30.0 ± 2.82	135.7	195.0
Zano 20	0.2	-	1.07 ± 0.05	38.5 ± 1.95	30.7 ± 1.56	140.2	196.1
Zano 20	0.5	-	1.08 ± 0.12	38.8 ± 1.93	34.8 ± 1.21	139.2	195.5
Zano 20	1.0	-	1.01 ± 0,09	38.9 ± 0.90	31.0 ± 1.39	139.3	197.8
Zano 20	2.0	-	0.87 ± 0.03	33.2 ± 1.13	32.1 ± 3.48	138.2	195.2
Zano 20	0.2	Stearic acid	1.08 ± 0.04	39.3 ± 0.98	31.6 ± 2.15	138.7	197.7
Zano 20	0.5	Stearic acid	1.06 ± 0.04	39.6 ± 1.40	36.44 ± 3.26	134.7	197.6
Zano 20	1.0	Stearic acid	1.10 ± 0.07	39.9 ± 1.16	31.6 ± 1.50	139.0	197.9
Zano 20	2.0	Stearic acid	0.93 ± 0.04	33.2 ± 1.09	33.2 ± 1.71	136.5	197.2

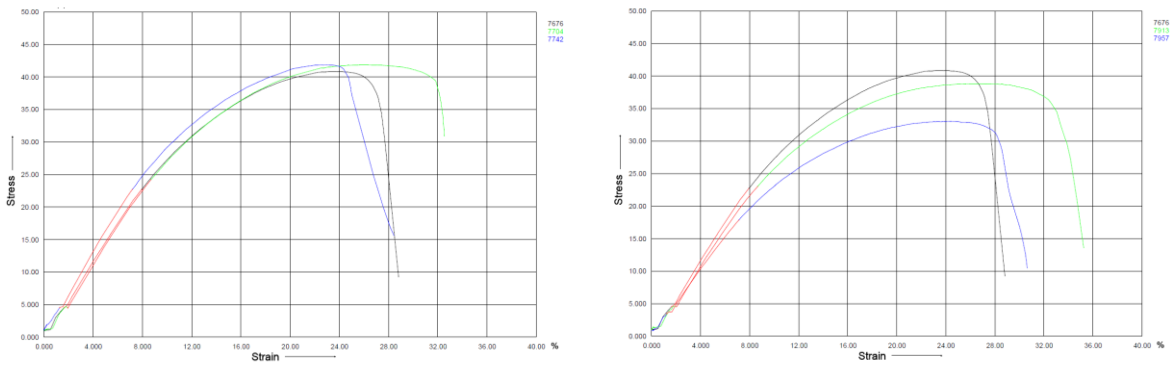

(A) (B)

Figure S2. Stress-strain curves of HDPE/ZnO composites as a function of ZnO concentration: 0 wt% - black; 0.5 wt% - green; 2.0 wt% blue: A) Zinkoxyd aktiv; B) Zano 20.

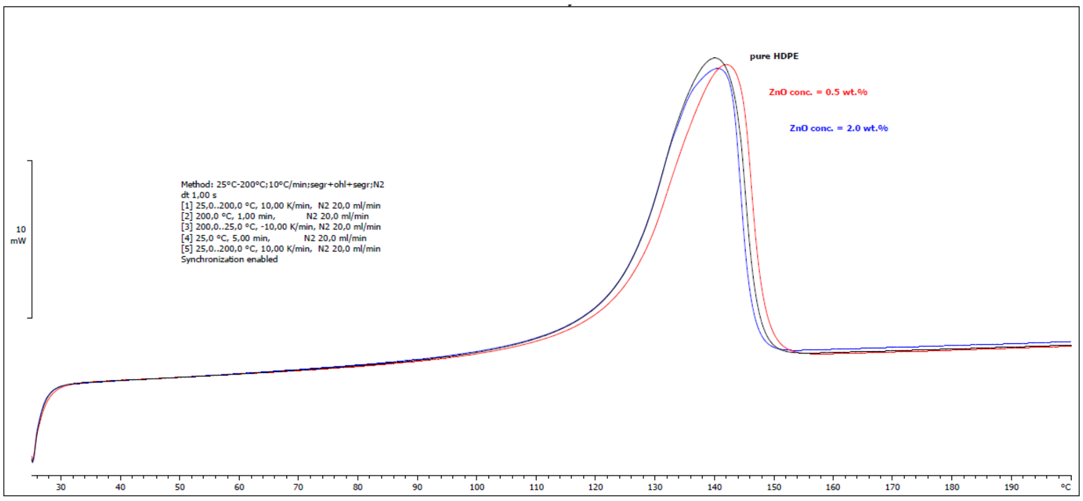

Figure S3. DSC curves of HDPE/ZnO composites as a function of ZnO concentration.

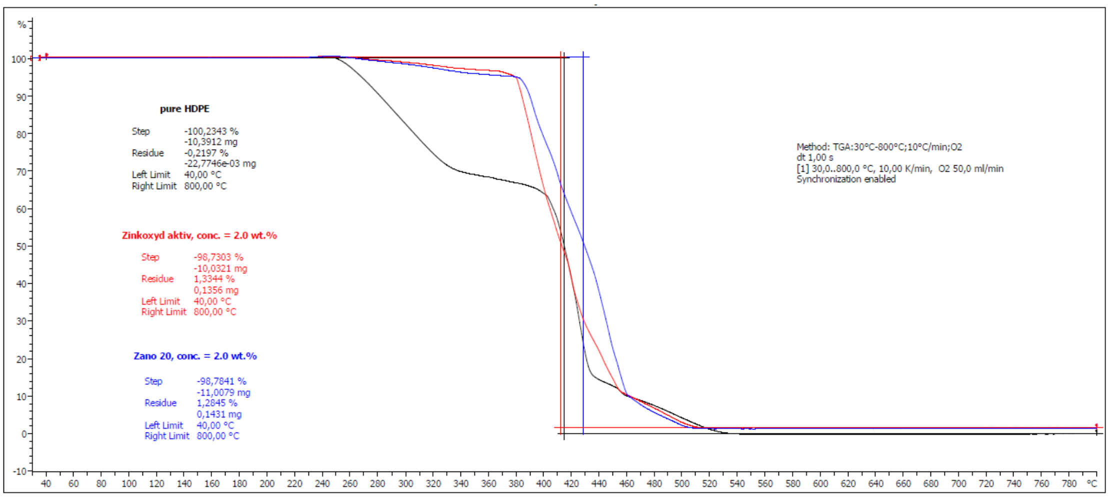

Figure S4. TGA curves of HDPE/ZnO composites as a function of ZnO concentration.

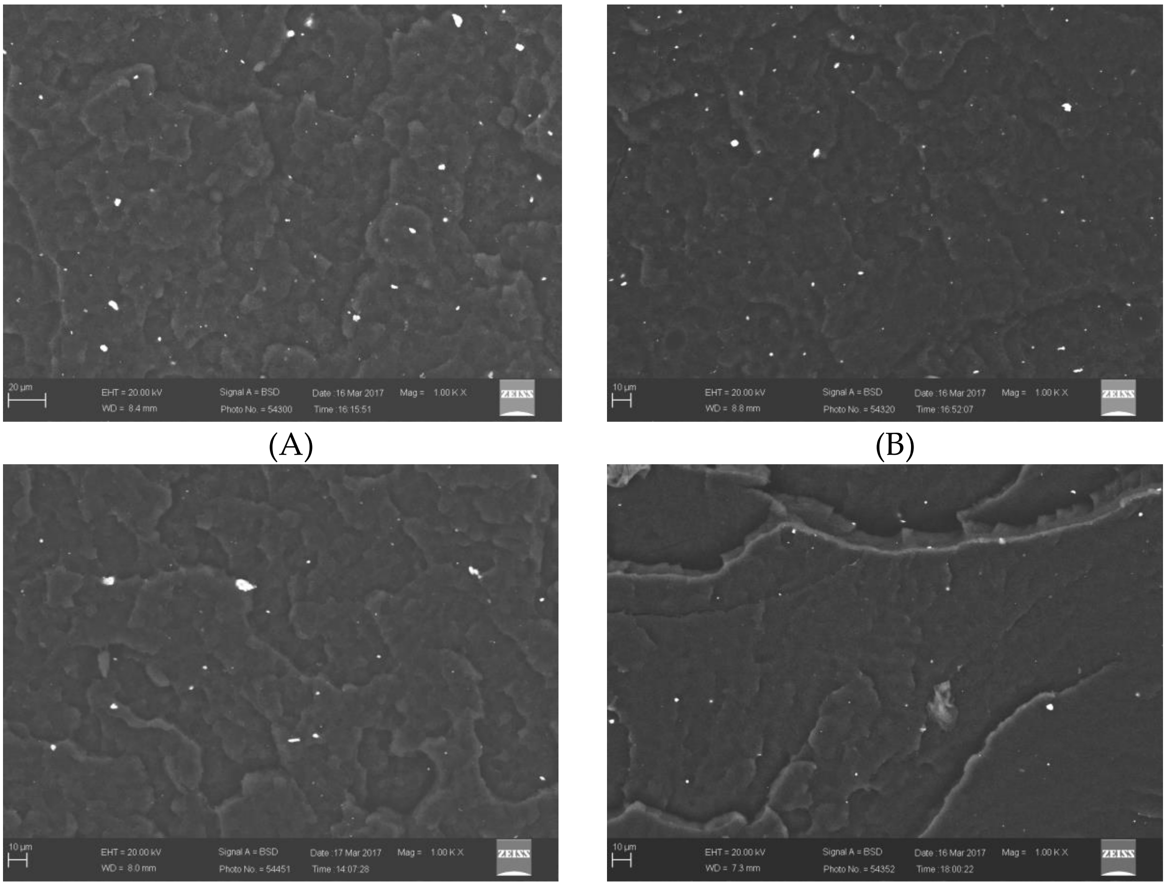

(A) (B) (C) (D)

Figure S5. SEM micrographs (magnification 1000×, backscattered electron detector) of PP/ZnO composites (Zinkoxyd aktiv – 1.0 wt%): A) unmodified nano ZnO - Zinkoxyd aktiv; B) nano ZnO - Zinkoxyd aktiv modified with stearic acid; C) unmodified nano ZnO - Zano 20; D) nano ZnO-Zano 20 modified with stearic acid.

Table S2. Mechanical and thermal properties of composites with PP matrix and ZnO - Zinkoxyd aktiv or Zano 20 (unmodified ZnO and surface modified with stearic acid) depending on the nano ZnO concentration. The polymer matrix is polypropylene PP - K499, the addition of stearic acid is 3.0 wt%, Znox-aktiv is Zinkoxyd aktiv.

**Type of ZnO**	**Conc. of ZnO wt%**	**Type of modification**	**Young's modulus GPa**	**Tensile strength MPa**	**Strain at break %**	**Melting temp. °C**	**ΔH_m J g^{-1}**
PP	0	-	1.26 ± 0.12	29.3 ± 0.41	69.5 ± 2.30	167.5	79.4
Znox-aktiv	0.2	-	1.31 ± 0.13	28.4 ± 0.83	58.1 ± 4.80	166.9	79.6
Znox-aktiv	0.5	-	1.27 ± 0.08	29.3 ± 0.58	56.0 ± 5.60	167.2	80.3
Znox-aktiv	1.0	-	1.32 ± 0.09	29.4 ± 0.38	58.4 ± 5.27	169.1	78.6
Znox-aktiv	2.0	-	1.32 ± 0.04	29.6 ± 0.59	57.1 ± 4.66	167.2	80.3
Znox-aktiv	0.2	Stearic acid	1.29 ± 0.07	28.9 ± 0.43	71.2 ± 4.25	166.7	80.0
Znox-aktiv	0.5	Stearic acid	1.25 ± 0.05	29.2 ± 0.35	58.7 ± 3.88	167.2	80.6
Znox-aktiv	1.0	Stearic acid	1.23 ± 0.03	28.9 ± 0.43	75.0 ± 4.11	168.1	78.2
Znox-aktiv	2.0	Stearic acid	1.23 ± 0.08	28.8 ± 0.50	57.8 ± 4.79	168.0	78.6
Zano 20	0.2	-	1.34 ± 0.12	31.4 ± 0.65	78.4 ± 4.58	166.8	79.3
Zano 20	0.5	-	1.30 ± 0.13	31.3 ± 0.41	73.1 ± 3.47	166.2	80.4
Zano 20	1.0	-	1.27 ± 0.09	31.2 ± 0.31	77.5 ± 4.44	167.4	78.0
Zano 20	2.0	-	1.30 ± 0.06	31.4 ± 0.63	79.7 ± 4.97	167.6	78.1
Zano 20	0.2	Stearic acid	1.29 ± 0.03	32.1 ± 0.66	77.8 ± 6.62	168.0	79.9
Zano 20	0.5	Stearic acid	1.28 ± 0.13	31.7 ± 0.58	69.5 ± 3.70	168.2	79.3
Zano 20	1.0	Stearic acid	1.29 ± 0.13	31.7 ± 0.70	79.6 ± 5.63	167.0	79.3
Zano 20	2.0	Stearic acid	1.21 ± 0.10	30.9 ± 0.62	69.4 ± 3.71	169.2	78.9

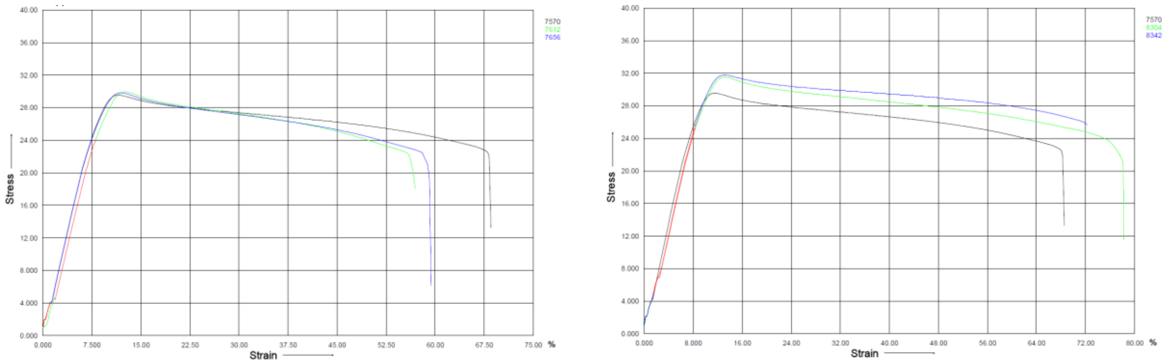

(A) (B)

Figure S6. Stress-strain curves of PP/ZnO composites as a function of ZnO concentration: 0 wt% - black; 0.5 wt% - green; 2.0 wt% blue: A) Zinkoxyd aktiv; B) Zano 20.

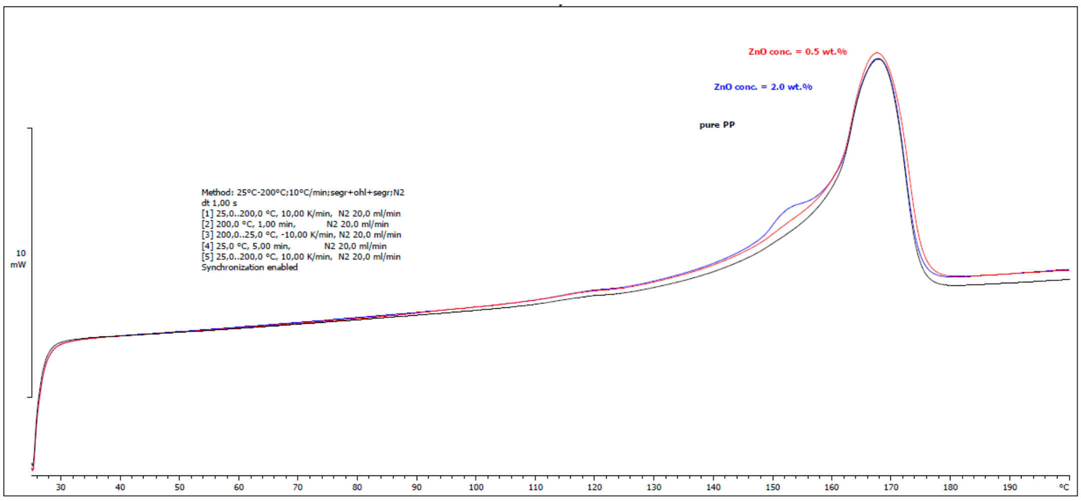

Figure S7. DSC curves of PP/ZnO composites as a function of ZnO concentration.

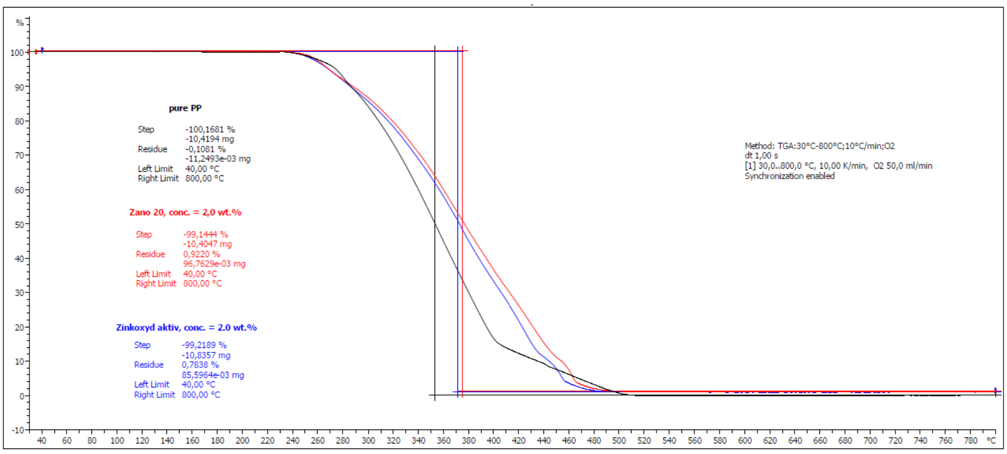

Figure S8. TGA curves of PP/ZnO composites as a function of ZnO concentration. .

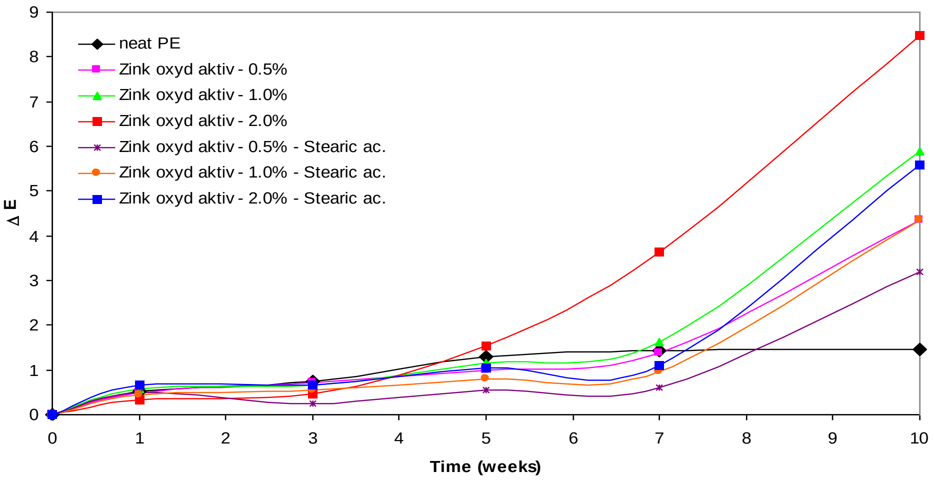

Figure S9. Changes in color of HDPE/ZnO composites (Zinkoxyd aktiv) as the functions of exposure time to artificial sunlight, nano ZnO concentration, and ZnO surface modification with stearic acid.

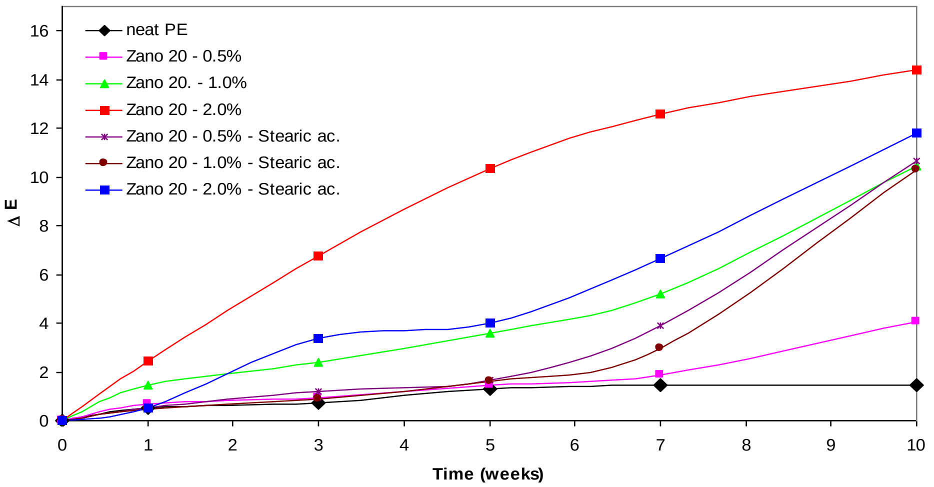

Figure S10. Changes in color of HDPE/ZnO composites (Zano 20) as the functions of exposure time to artificial sunlight, nano ZnO concentration, and ZnO surface modification with stearic acid.

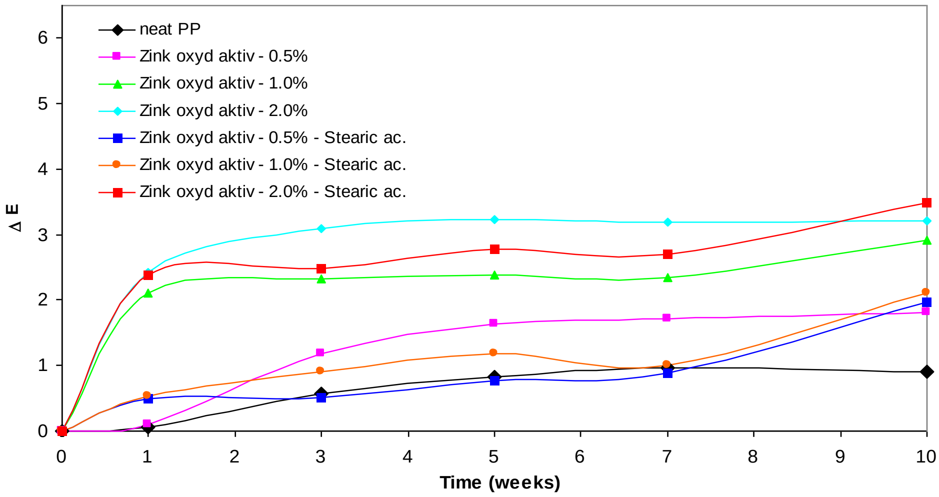

Figure S11. Changes in color of PP/ZnO composites (Zinkoxyd aktiv) as the functions of exposure time to the artificial sunlight and ZnO concentration.

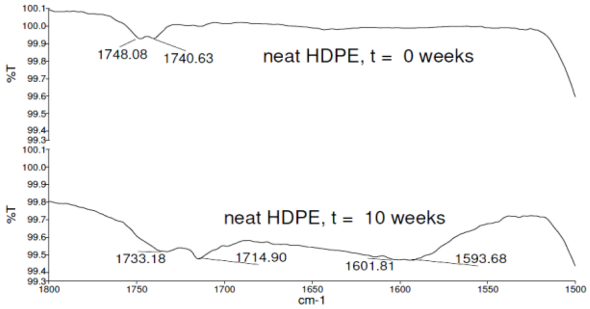

(A)

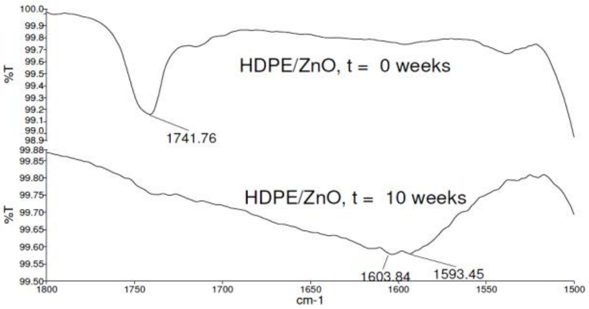

(B)

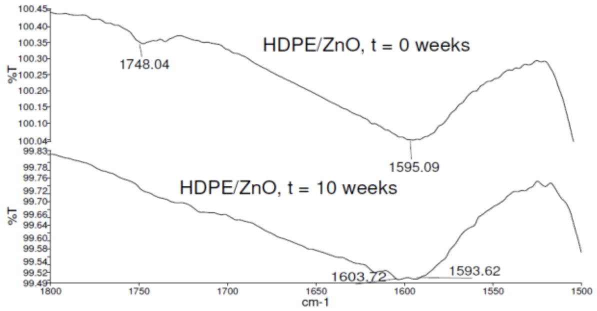

(C)

Figure S12. FTIR spectra of HDPE/ZnO composites prior to exposure to artificial light and after ten weeks of exposure: A) pure HDPE; B) HDPE with 1.0 wt% of ZnO (Zinkoxyd-aktiv); C) HDPE with 1.0 wt% of ZnO (Zano 20).

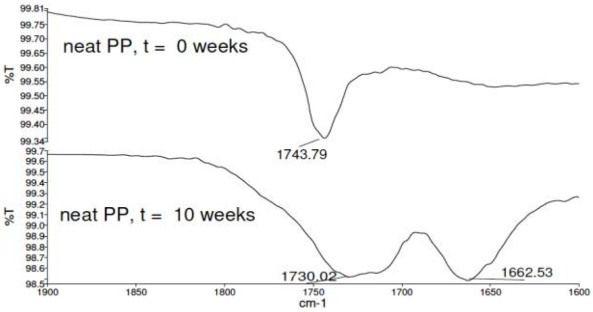

(A)

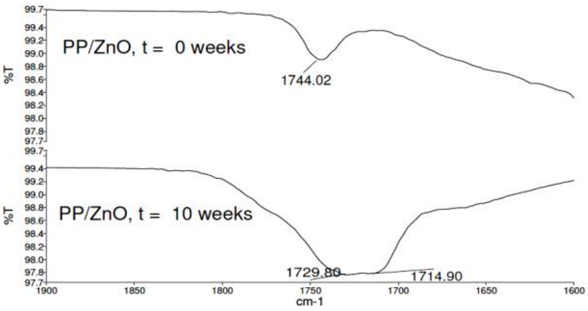

(B)

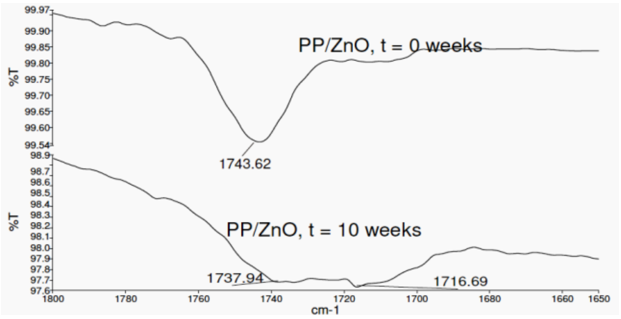

(C)

Figure 13. FTIR spectra of PP/ZnO composites prior to exposure to artificial light and after ten weeks of exposure: A) pure PP; B) PP with 1.0 wt% of ZnO (Zinkoxyd-aktiv); C) PP with 1.0 wt% of ZnO (Zano 20).

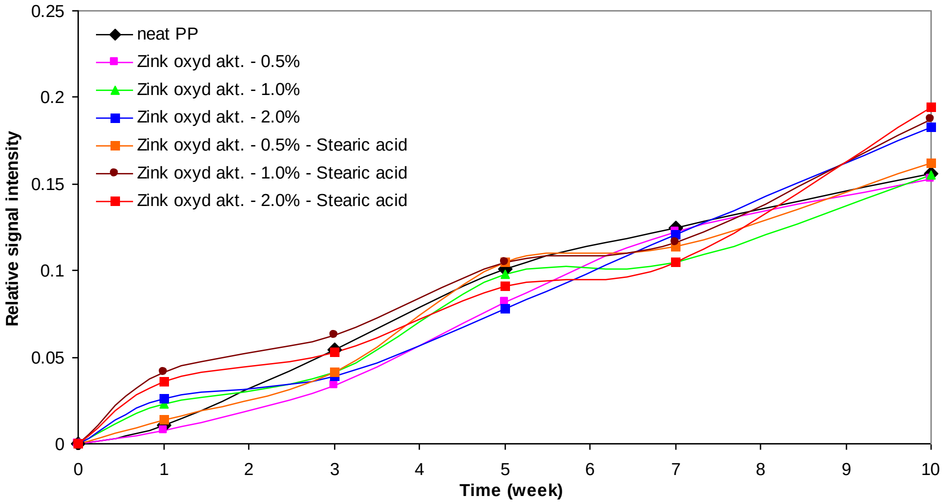

Figure S14. Intensity of the absorption band at 1725 cm^{-1} in PP/ZnO composites as a function of exposure time to the artificial sunlight.

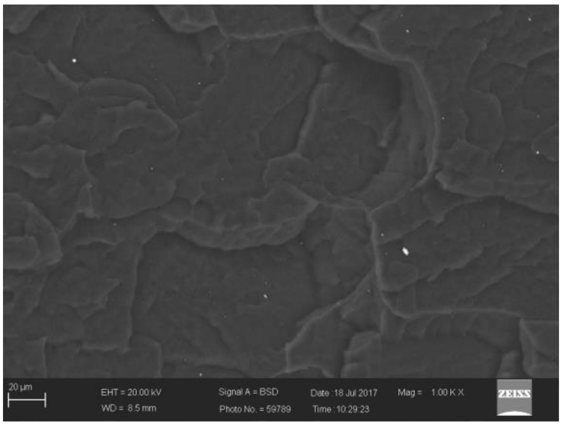

(A)

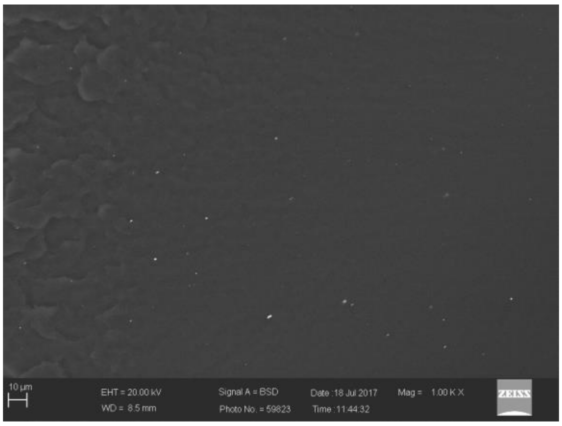

(B)

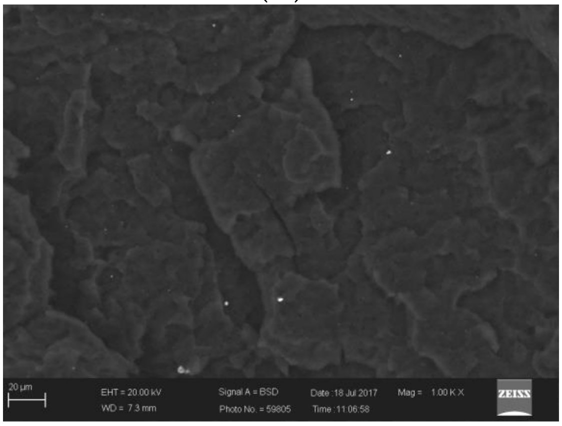

(C)

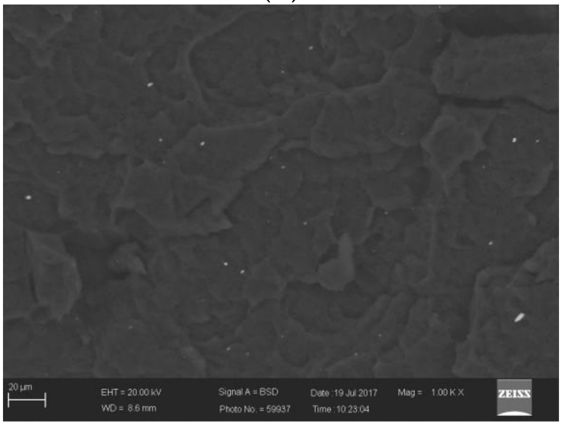

(D)

Figure S15. SEM micrographs (magnification 1000×, backscattered electron detector) of HDPE/ZnO composites with 1.0 wt% of silane modified ZnO: A) Zano 20 Plus; B) Zano 20 Plus 3, and PP/ZnO composites: C) Zano 20 Plus; D) Zano 20 Plus 3.

Table 3. Mechanical and thermal properties of composites of HDPE matrix and silanized nano ZnO (Zano 20 Plus - 3.9 wt% of caprylyl silane and Zano 20 Plus 3 – 1.0 wt% of methacrylic silane).

Type of ZnO	Conc. of ZnO wt%	Young's modulus GPa	Tensile strength MPa	Strain at break %	Melting temp. °C	ΔH_m J g^{-1}
HDPE	0	1.16 ± 0.10	41.9 ± 1.31	28.3 ± 3.07	136.8	197.2
Zano 20 Plus	0.2	1.08 ± 0.10	43.6 ± 1.27	30.5 ± 2.87	135.1	196.3
Zano 20 Plus	0.5	1.07 ± 0.07	43.9 ± 0.39	30.0 ± 1.66	136.4	197.6
Zano 20 Plus	1.0	1.10 ± 0,10	43.7 ± 0.89	32.0 ± 2.32	134.5	196.5
Zano 20 Plus	2.0	1.17 ± 0.07	42.9 ± 0.68	31.7 ± 2.43	135.2	195.5
Zano 20 Plus 3	0.2	1.18 ± 0.03	40.6 ± 1.33	32.4 ± 0.70	135.7	198.0
Zano 20 Plus 3	0.5	1.19 ±0.07	41.7 ± 0.89	29.6 ± 2.41	135.3	196.5
Zano 20 Plus 3	1.0	1.12 ± 0.06	41.0 ± 0.78	31.6 ± 2.79	134.4	196.1
Zano 20 Plus 3	2.0	1.13 ± 0.09	41.3 ± 1.41	30.7 ± 3.14	135.8	194.1

Table 4. Mechanical and thermal properties of composites with PP matrix and silanized nano ZnO (Zano 20 Plus – 3.9 wt% of caprylyl silane and Zano 20 Plus 3 – 1.0 wt% of methacrylic silane).

Type of ZnO	Conc. of ZnO wt%	Young's modulus GPa	Tensile strength MPa	Strain at break %	Melting temp. °C	ΔH_m J g^{-1}
PP	0	1.26 ± 0.12	29.3 ± 0.41	69.5 ±2.30	167.5	79.4
Zano 20 Plus	0.2	1.21 ± 0.07	31.6 ± 0.31	75.6 ± 4.78	168.2	90.8
Zano 20 Plus	0.5	1.21 ± 0.09	31.9 ± 0.53	93.6 ± 3.67	166.7	90.4
Zano 20 Plus	1.0	1.18 ± 0.11	31.2 ± 0.79	73.6 ± 7.42	166.3	89.9
Zano 20 Plus	2.0	1.14 ± 0.10	31.1 ± 1.33	66.9 ± 2.37	168.0	89.6
Zano 20 Plus 3	0.2	1.27 ± 0.06	30.1 ± 0.04	72.8 ± 6.98	167.3	79.2
Zano 20 Plus 3	0.5	1.18 ± 0.04	29.3 ± 0.59	67.8 ± 6.37	168.5	77.4
Zano 20 Plus 3	1.0	1.20 ± 0.10	29.0 ± 0.33	66.4 ± 3.11	167.2	78.8
Zano 20 Plus 3	2.0	1.29 ± 0.04	30.4 ± 0.40	87.4 ± 6.41	167.1	78.1

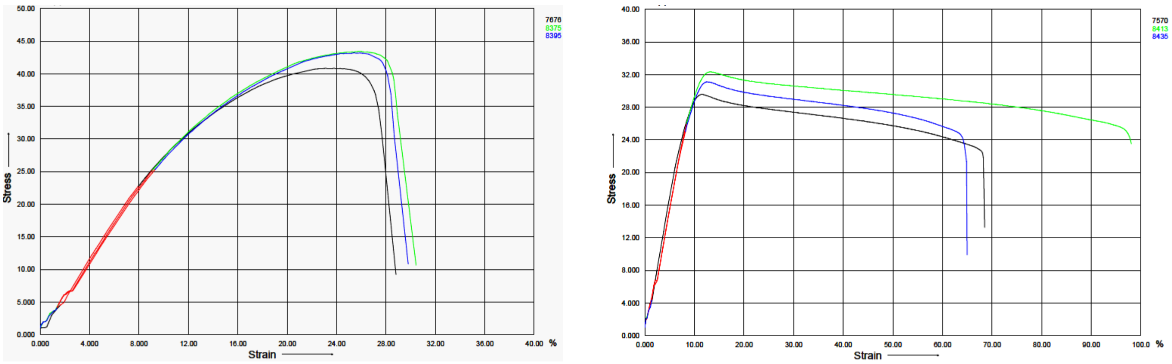

(A) (B)

Figure S16. Stress-strain curves of polyolefin/ZnO composites as a function of silanized ZnO concentration (Zano 20 Plus): 0 wt% - black; 0.5 wt% - green; 2.0 wt% blue. A) HDPE and B) PP.

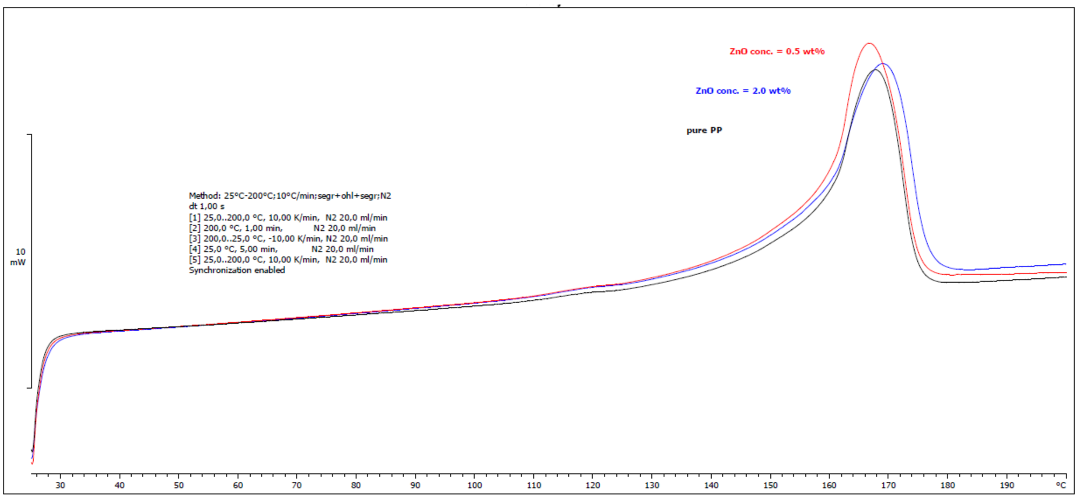

Figure S17. DSC curves of PP/ZnO composites as a function of silanized ZnO concentration (Zano 20 Plus).

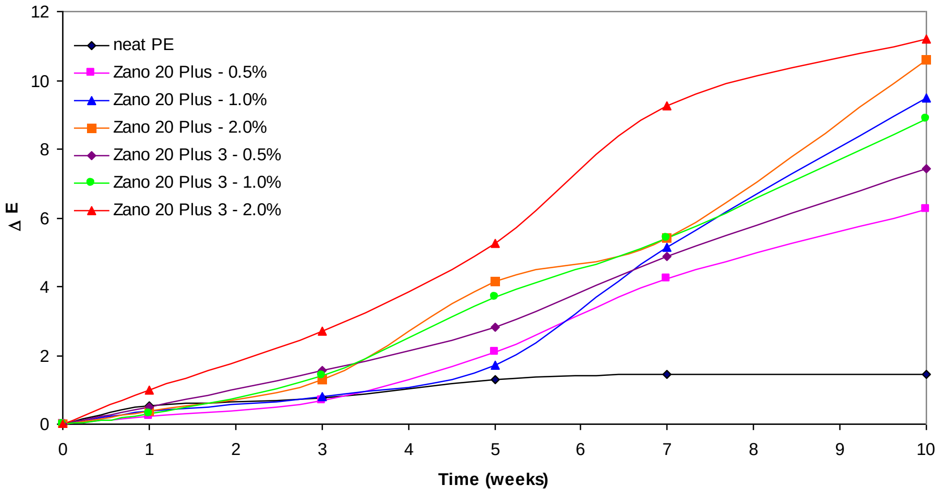

Figure S18. Change in color of HDPE/ZnO composites (Zano 20 Plus and Zano 20 Plus 3) depending on the exposure time to artificial sunlight and nano ZnO concentration as well as the type of surface modification.

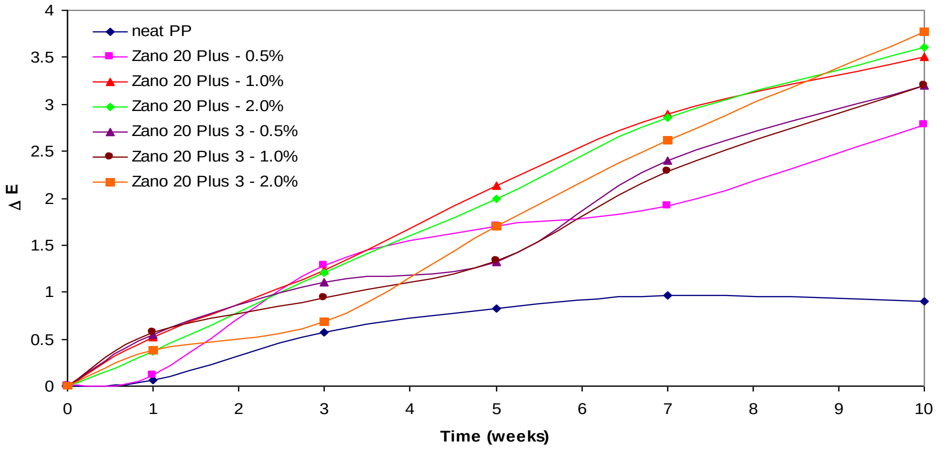

Figure S19. Change in color of PP/ZnO composites (Zano 20 Plus and Zano 20 Plus 3) as the functions of the exposure time to artificial sunlight and nano ZnO concentration as well as the type of surface modification.
